# Cardiac biomarkers and ultrasonography as tools in prediction and diagnosis of traumatic pericarditis in Egyptian buffaloes

**DOI:** 10.14202/vetworld.2016.976-982

**Published:** 2016-09-16

**Authors:** Noura E. Attia

**Affiliations:** Department of Animal Medicine, Faculty of Veterinary Medicine, Zagazig University, Egypt

**Keywords:** buffaloes, cardiac biomarkers, diagnosis, traumatic pericarditis, ultrasonography

## Abstract

**Aim:**

This study was designed to evaluate the cardiac biomarkers and ultrasonography in prediction and early diagnosis of traumatic pericarditis (TP) in Egyptian buffaloes.

**Materials and Methods:**

A total number of 47 buffaloes were included in the study and divided into two groups: Healthy (n=10) and diseased groups (n=37). Diseased buffaloes were admitted to the Veterinary Teaching Hospital at Zagazig University, Egypt, with a history of anorexia, sudden, and severe reduction of milk production with no response to a previous medical treatment some animals had edema at the dewlap and congestion of the jugulars. These animals were subjected to clinical examination, evaluation by hemato-biochemical analysis including cardiac biomarkers and sonography.

**Results:**

The hemato-biochemical analysis revealed leukocytosis with a shift to left and hyperfibrinogenemia (indicating inflammation). Serum cardiac biomarkers including cardiac troponin I (cTnI), cTnT, nitric oxide, creatine kinase myocardial band, and lactic dehydrogenase enzyme were significantly increased in buffaloes with TP compared with control ones. Ultrasonographically, there were hypoechoic materials with echogenic fibrin interspersed in between the pericardial sac.

**Conclusions:**

The cardiac biomarkers may be considered a useful index in the early diagnosis of TP. Moreover, ultrasonography is an excellent tool for early prediction and diagnosis of such condition.

## Introduction

In the veterinary field, traumatic pericarditis (TP) is one of the most important cardiac diseases among bovines. It considered a frustrating problem causing devastating economic losses due to the sudden and sharp decrease of milk production, treatment costs, and finally losses of the animal as usually is associated with progressive disturbances in heart function [1]. In bovines, pericarditis is defined as inflammation of the pericardium with the accumulation of large amounts of serous or fibrinous inflammatory products in the pericardial sac [2]. It is commonly occurring as a consequence of traumatic reticuloperitonitis.

The earliest clinical sign of TP is tachycardia. However, this sign is not specific as it may present in many physiological and other pathological conditions. Hence, the early diagnosis of such condition is difficult as the most cases present in advanced stages when the cardiac dysfunction produce clinical signs of heart failure which had a poor prognosis [3]. Diagnosis of advanced cases based on clinical examination as pathognomonic clinical signs “including bilateral jugular venous distention and/or pulsation and edema at dewlap” are characteristic for the diseased condition [4]. Although, many cases do not show all of these characteristic signs and diagnosis may therefore not be straight forward as pathognomonic signs may appear in other diseases [2]. Hence, case history and clinical examination are not sufficient for the diagnosis of TP. Thus, additional diagnostic aids are required for early prediction and diagnosis of TP as cardiac biomarkers and ultrasonographic examination. Cardiac biomarkers including cardiac troponins (cTn), nitric oxide (NO), the cardiac origin of creatine kinase ‘creatine kinase myocardial band’ (CK-MB), and lactic dehydrogenase enzyme (LDH).

Troponin is a globular protein localized on thin filaments of striated muscle and consists of three subunits; Tn-I, Tn-T, and Tn-C [5,6]. cTnI was an excellent candidate biomarker of cardiac injury in all mammals [7]. It is used as a highly specific marker of myocardial damage [8], as it released into the circulation during myocardial cell damage [9]. cTnT is a cardiac structural, regulatory protein. It is used clinically as a highly specific marker of myocardial damage, so used in the diagnosis of acute myocardial infarction and cardiovascular diseases [10]. NO is produced from all cell types composing the myocardium and regulates cardiac function via autonomic control mechanism and it has an important role in the suppression of arrhythmias. The deficiency of endothelial NO synthase predisposes to the occurrence of cardiac arrhythmias [11]. Hence, NO has an important role in the heart scan. Ultrasonography has been reported as a non-invasive imaging modality for diagnosis and prognosis of TP [12,13].

The bovine cardiac disease has received little clinical attention as the early diagnosis is not frequently made leading to progress of the condition. Thus, this study was designed to throw light on the uses of cardiac biomarker and ultrasonography in the early diagnosis of TP in buffaloes.

## Materials and Methods

### Ethical approval

This study was approved by the Institutional Animal Ethics Committee.

### Animals

A total of 37 buffaloes (*Bubalus bubalis*), 20 buffalo was pregnant over 9 months and 12 animals were recently calved since 1 month, and 5 animals were nonpregnant were admitted to the Veterinary Teaching Hospital at Zagazig University, Egypt, with a history of anorexia and decrease of milk production. Animals were with a past history of receiving of various medical treatments with no response. Clinical signs appeared on animals vary from only tachycardia, reluctance to walk or walk with short steps with abducted elbows. In other cases, there was bilateral venous distension and sometimes pulsation, edema of dewlap and the ventral abdominal region extending up to the udder. Deep palpation of the ventral abdominal wall behind the xiphoid commonly elicits a painful grunt. The mucous membranes may be congested and have a prolonged capillary refill time. In addition, 10 clinically healthy animals were included in this study as a control group.

### Clinical assessment

All animals were subjected to thorough clinical examination according to the method of Jackson and Cockcroft [14]. In addition, examination of the jugular and milk veins and pain tests were applied.

### Blood sampling

About 10 ml of blood was collected from all investigated buffaloes by jugular vein puncture, 2 ml of blood was transferred into vacuum ethylenediaminetetraacetic acid coated tubes for hematological examination including “hemoglobin, packed cell volume, total erythrocyte count, total leukocyte count, and differential leukocyte count” which were estimated within 2-4 h of collection using the blood cell counter machine. The remaining blood samples were divided then added to heparinized tubes and tubes without anticoagulant to yield plasma and serum, respectively. Blood plasma and serum samples were harvested by centrifugation at 3000 rpm for 10 min, and then kept frozen at −20°C until the further analysis [15]. Fibrinogen (Fb) was estimated in plasma samples using commercially available bovine kits according to Orro *et al*. [16].

### Biochemical analysis

Serum total proteins, albumin, globulin, and liver enzymes (aspartate aminotransferase [AST] and alanine aminotransferase [ALT]) were measured by standard procedures using Diagnostic Zrt. Commercial kits which were provided by Biomerieux, Egypt, and the reading was taken by spectrophotometer. The activities of CK-MB and LDH were measured spectrophotometrically with commercial kits. The concentration of cTnI was determined in samples of serum with a commercial kit (Card-I-kit Combo Test; AboaTech). The test was carried out according to the manufacturers’ instructions. While cTnT was measured quantitatively using electrochemiluminescence technology 3 generation cTnT (Roche Diagnostics, Mannheim, Germany) [17]. Estimation of serum NO concentration was carried out according to Beda and Nedospasov [18].

### Ultrasonographic examination

The area over the heart and reticulum of the left and right sides of the thorax (3^rd^-7^th^ intercostal space) up to the level of the elbow was examined using 3.5 MHz convex transducer. Animals were secured in standing position without sedation. The reticulum and surrounding tissues were examined as described by Braun and Goetz [19]. The probe was placed first at the area just behind the xiphoid cartilage then moved caudally and laterally to assess reticular shape, contour, and motility per 2 min. The heart was examined as described by Braun [1]; both sides of the cardiac area were scanned by 3.5 MHz sector probe. Examined area was clipped with adding of coupling gel to facilitate the examination.

### Statistical analysis

The results data were presented as the mean±standard error of mean. The groups were compared by independent sample t-test in IBM SPSS Statistics version 21. The statistical significance of the parameters was determined in the tests at p<0.05.

## Results

Age of affected animals was ranged from 4 to 9 years old. As regard to the sex of the affected buffaloes, the disease is a more predominant in females than males. The clinical signs shown by the diseased animals differed because of variation in the severity of the disease and the extent of the lesions as in Table-1. Some animals only exhibit moderately to severely tachycardia “10 cases.” In these cases, there was neither brisket edema nor congestion of the jugular vein “pathognomonic signs of TP.” Other diseased conditions showed abdominal respirations, arched back with abducted elbows, ventral edema which is the most common symptom associated with pericarditis and bilateral jugular venous distension/pulsation.

**Table-1:** Clinical findings in clinically healthy buffaloes and those with TP (excluding cardiac signs).

Variable	Control buffaloes	Buffalo with TP	Number of affected buffaloes
General condition	Normal “alert”	Mild depression	7
		Moderately depressed	20
		Severely depressed	10
Appetite	Normal	Decreased	15
		Anorexia	22
Posture	Normal	Normal	14
		Arched back	8
		Abducted elbow	15
Characters of feces	Semi-solid dark green flat cakes	Intermittent diarrhea	10
		Firm scanty feces	27
Pain test	Negative (no painful reaction)	Negative	10
		Positive (painful grunting)	27
Metalic detector	Negative	Negative	12
		Positive	25
Skin turgor	Normal	Normal	10
		Decreased	27

TP=Traumatic pericarditis

Alteration in physiological parameters was shown in Table-2, the body temperature was significantly increased, only decreased in the very advanced cases. In our study, auscultation of the heart revealed mainly tachycardia which was the only abnormal findings on auscultation in some cases (7 cases). Frictional or rubbing sound can detect in 3 cases only, muffled sound in 18 cases, and splashing in 9 cases.

**Table-2:** Cardiovascular findings and physiological parameters in clinically healthy buffaloes and those with TP.

Variable	Control buffaloes	Buffaloes with TP	Number of affected buffaloes
Edema	Non	Non	10
		Brisket	26
		Throat region	1
Jugular veins	Normal	Normal	15
		Distension	22
		Distension/pulse	10
Heart rate	Normal 72.5±2.28	Normal	5
		Tachycardia (92.35±2.03)	32
Pericardial sounds	Non	Non	7
		Frictional	3
		Muffled	18
		Present “tinkling-splashing”	9
Rectal temperature (°C)	38.4±07	Increased	38.8±0.09*
		Decreased	37.8±0.04*
Respiration rate “breathe/minute”	21.5±1.24	Increased	25.5±0.79*
Ruminal motility/2 min	3.1±0.2	Decreased	0.55±0.1**
		Absent	

* Significant at p<0.05,

** Significant at p<0.01. TP=Traumatic pericarditis

The values of hematological parameters revealed an elevated total leukocyte count (p<0.01) with neutrophilia and lymphocytopenia (Table-3) as compared to the control group. Affected buffaloes showed hyperfibrinogenemia with Fb concentrations >800 mg/dL.

**Table-3:** Mean values (±SE) of hematological and biochemical indices in clinically healthy buffaloes and those with TP.

Parameters (Mean±SE)	Control buffaloes	Buffaloes with TP
Hb (g%)	10.8±0.25	10.1±0.1*
PCV (%)	28.7±0.7	33.9±0.9**
TEC (×10^6^/mL)	6.28±0.24	5.07±0.23*
TLC (×10^3^/mL)	6.24±0.14	12.75±0.68**
Neutrophils (%)	30.67	46.33**
Lymphocytes (%)	49.54	30.7**
Eosinophils (%)	2.96	0.52**
Fibrinogen (mg/dl)	345±58.7	848.69±109.9**
Total proteins (g/dl)	7.22±0.24	6.28±0.21*
Albumin (g/dl)	3.9±0.15	2.27±0.07**
Globulin (g/dl)	3.3±0.23	4.2±0.15**
AST (U/L)	91.44±5.04	412.5±13.1**
ALT (U/L)	30.05±2.2	44.85±1.07**
CTnI (ng/mL)	0.029±0.003	2.31±0.48**
CTnT (ng/mL)	0.093±0.018	0.632±0.22*
NO (μM)	2.28±0.19	3.47±0.22**
CK-MB (U/L)	131.74±1.87	199.71±6.93**
LDH (U/L)	515.75±1.87	750.73±65**

* Significant at p<0.05,

** Significant at p<0.01. Hb=Hemoglobin, PCV=Packed cell volume, TEC=Total erythrocyte count, TLC=Total leucocyte count, AST=Aspartate aminotransferase, ALT=Alanine aminotransferase, CTnI=Cardiac troponin I, NO=Nitric oxide, CK-MB=Creatine kinase myocardial band, LDH=Lactic dehydrogenase enzyme

The alterations in biochemical parameters were given in Table-3. As collected in the table, the levels of serum total proteins and albumin were significantly decreased with a significant increase in globulin. The cardiac biomarkers concentration was significantly increases in all diseased buffaloes even those animals which do not exhibit the pathognomonic signs of TP at the time of presentation.

### Ultrasonographic observations

**Figure 1:**
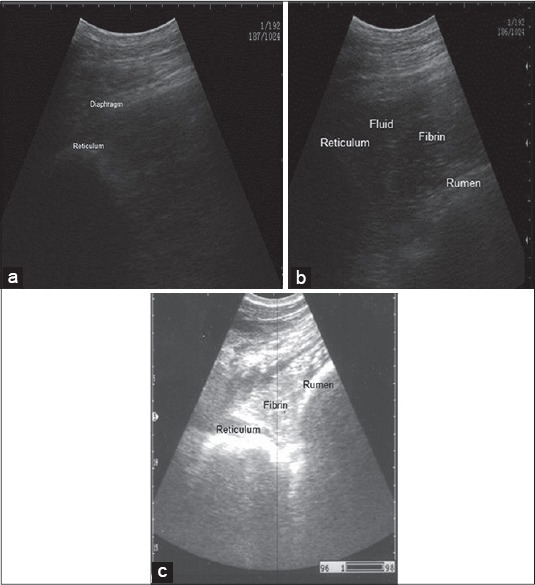
Ultrasonographic image of normal reticulum, (a) notice half-moon shape of the wall, imaged from ventral midline of abdomen, left 7^th^ intercostal space (ICS). Deposition of the echogenic fibrin interspersed with the hypoechogenic fluid between the reticulum and cranial sac of the rumen. Imaged from 7^th^ ICS at the midline (b and c), notice, corrugated hyperechogenicity of the reticular wall (c).

**Figure 2:**
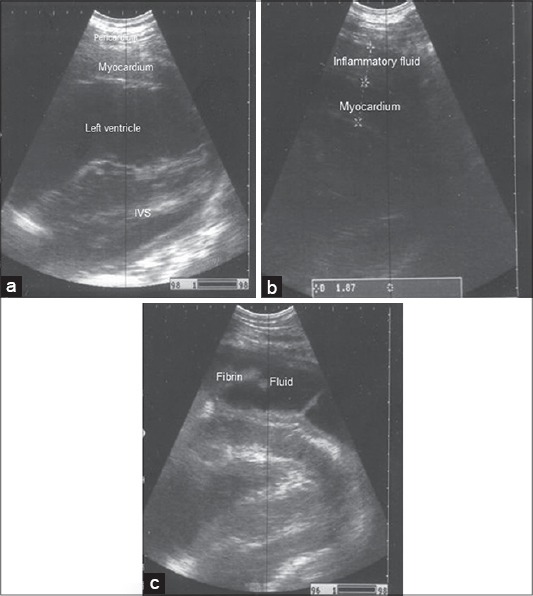
Ultrasonographic image of normal heart, (a) Notice, the narrow distance between visceral and parietal pericardium, IVS “interventricular septum.” Small amount of anechoic pericardial effusion and echogenic fibrin at the pericardial sac. Imaged at 4^th^ intercostal space (ICS), (b) increase the distance between the pericardium and epicardium by a large amount of anechoeic pericardial effusion and hyperechoic fibrin at the pericardial sac, (c) imaged at 4^th^ ICS.

**Figure 3:**
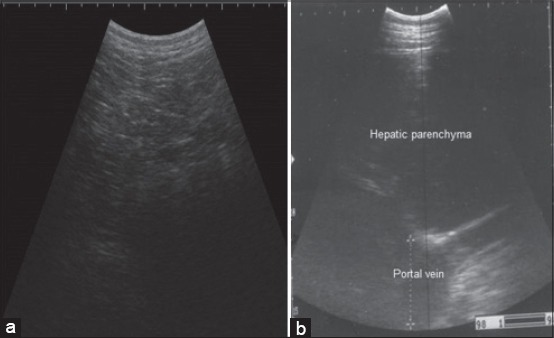
Ultrasonographic imaging of presternal edema in a buffalo with traumatic pericarditis where an excessive accumulation of anechoic fluid separated with echogenic septae is present, (a) ultrasonogram showing dilated portal vein (b) obtained from the 10^th^ intercostal space.

Ultrasonographic examination of healthy bovine reticulum revealed a half moon shaped with a smooth contour as in Figure-1a that contract at regular intervals “2-3 contraction/2 min.” Reticular contents are gaseous, thus cannot normally be visualized sonographically. While in all diseased buffaloes, the reticulum appeared moderately to severely corrugated, a tonic “either reticular motility is decreased or completely absent.” Ultrasonography of the abdomen revealed reticular changes with a large amount of fibrin debris interspersed with fluid pockets were frequently seen between the reticulum and dorsal ruminal sac as in Figure-1b and c. Cardiac ultrasonography revealed thick hyperechogenic pericardium and epicardium with increase distance between as in Figure-2b and c. On contrary to the normal cardiac image as small distance between the pericardium and epicardium as in Figure-2a. Ultrasonographic imaging of the presternal edema revealed excessive accumulation of anechoic fluid separated with an echogenic septae (Figure-3a). There was a dilation of the portal vein as in Figure-3b in the diseased buffaloes.

## Discussion

TP is the most common disease as a consequence of traumatic penetration of the pericardium by a foreign body arising from the gastrointestinal tract. Incidence of the disease was higher in females, and this may be attributed to the pressure of pregnancy and effort of parturition which gives the chance to push the foreign body forward causing penetration of the pericardium.

The lack of the pathognomic signs of TP in some cases may be attributed to compensation by the cardiac reserve. These compensatory responses include the redistribution of the blood flow and increased heart rate and its contractility [4]. Clinical signs exhibited by the animals were similar to that previously described by Braun *et al*. [20] and Ghanem [21]. Engorgement of jugular vein and brisket edema may be attributed to the pericardial effusion as the volume of fluid in the pericardial sac increased, increase the intracardiac pressure, constrain the cardiac output, impedes the venous return to the heart, increase the venous hydrostatic pressure causing the ventral edema, and right-sided cardiac insufficiency and congestive heart failure which usually seen on terminal stages of the disease [1].

The significantly higher rectal temperature was observed, indicating systemic reaction similar findings reported by Ghanem [21] and this may be due to toxemia in the early stage as endotoxins lead to the release of inflammatory cytokines causing the pyrexia [4]. However, moderate hypothermia was recorded in the late stages of the disease, our results agreed with those obtained by Sharma *et al*. [22]. The significant increase in the respiratory rate indicates respiratory distress associated with toxemia and septicemia caused by the foreign body penetration or due to cardiac insufficiency or direct involvement of the lungs [21].

Heart sound detected on TP varies depending mainly on the type of lesion [2]. The severity of tachycardia depends primarily on the degree of compression of the heart by pericardial effusion [1]. In the early stage, frictional or rubbing sound can detect as parietal and visceral pericardium rub against each other and this sound was heard in 3 cases only as the most cases of heart diseases are present in advanced stages. In advanced cases muffled heart sound heard and this may be attributed to pericardial effusion and fibrin changes. When the changes are mainly fibrin, the sounds are of a scratching in nature. While if the fluid was predominant, there are splashing or gurgling sounds, similar results previously described by Braun *et al*. [20].

The marked leukocytosis may be attributed to the infection associated with the penetration of the reticulum and diaphragm by a foreign body. This indicates the presence of large amounts of pus or characteristics of a large internal abscess, which induces a more severe inflammatory response [4,23]. In contrast, there was lymphocytopenia and eosinopenia, which might due to a reduction in cellular immunity caused by the stress of penetration [4]. Hemoconcentration which recorded may be due to dehydration or toxemia.

Buczinski *et al*. [23] and Ducharme and Fubini [24] recorded that cattle with TP had a hyperfibrinogenemia. Fb may be the best indicator of acute inflammation because Fb concentrations often increase before the development of neutrophilia [25]. As its elevation indicates the severity of the inflammatory nature of the disease. Fb concentration is preferable as a marker as compared to the leukogram when evaluating traumatic inflammatory processes [26].

Hypoproteinemia, hypoalbuminemia with hyperglobulinemia which recorded in diseased buffaloes were previously described by Buczinski *et al*. [23] and Saleh *et al*. [27] and this may be attributed to the hepatic dysfunction. Low concentrations of albumins together with Fb levels confirmed a severe inflammatory state. The increased activities of AST and ALT could be attributed to the hepatic congestion and not primary liver disease [1], similar results recorded by Sharma *et al*. [22].

The significant increase of cTnI (p<0.01) and cTnT (p<0.05) concentration in TP affected group indicates myocardial cell damage [28] as cTn proteins, which are usually present in blood either at very low concentrations or below the limit of detection of most assays, are released into the circulation in pericarditis. The results of this study indicate that cTnI and cTnT are useful diagnostic aids for the confirmation of pericarditis in buffaloes especially those without pathognomonic signs “engorgement of jugular vein and brisket edema.” Their elevated levels considered a reflection of myocardial damage and cardiac insufficiency [10].

The significantly high value of NO (p<0.01) in diseased buffaloes may be attributed to defensive purposes and inflammatory process, similar results obtained by Rakhit *et al*. [11]. A significant increase (p<0.01) of CK-MB and LDH in our study can be used as an indicator of myocarditis in which the release of theses enzyme is higher than expected when myocardial cells damaged. CK-MB is a subunit of CK which rises if the heart muscle damaged [29]. On the other hand, it has been demonstrated that the LDH level increases after an injury to the liver, skeletal muscle, cardiac muscle, and kidney [29]. Hence, the significantly higher value of LDH might have been due to the severity of skeletal and cardiac muscle damage, in addition to liver involvement in the disease.

### Ultrasonographic observations

Ultrasonography is an integral part of recent bovine medicine. The ultrasonographic picture of the normal bovine reticulum was similar to those previously described by Braun and Goetz [19] and Braun *et al*. [30]. Reduction or completely absence of the reticular movement occurs due to adhesion with the abdominal wall. Ultrasonography of the abdomen revealed reticular changes typical to the findings of traumatic reticuloperitonitis [27]. Increase the distance between the pericardium and epicardium due to the presence of hypoechoic pericardial effusion “interspersed with echogenic deposits, representing fibrin strands.” This result is comparable to that obtained in other studies [12,30]. Hence, cardiac ultrasonography has been suggested as a reliable tool and a method of choice for imaging and evaluating the severity of TP. Ultrasonographic examination is a substantial aid in diagnosing congestion in the systemic circulation, which indicated by hepatic congestion as there is a dilation of the portal vein and this may be attributed to hydrostatic pressure in the portal vein secondary to pericardial effusion [31].

Prognosis is a poor as most animals in advanced stages had signs of congestive heart failure died within 1 or 2 weeks [32], so buffaloes with TP should be slaughtered rather than treated [1,20]. The treatment of pericarditis is often unrewarding and usually and is addressed toward salvaging or short-term survival to calving. Hence, the early diagnosis of the disease depending on cardiac biomarkers and ultrasonography is essential to reduce animal suffering and owner costs.

TP in bovines can be prevented to a large extent by the proper management manners and controlled by routine administration of magnets to heifers at the time of pregnancy diagnosis. The processed feed can be passed over magnets to detect any magnetic foreign bodies before being fed to the animal.

## Conclusion

As cattle, buffaloes kept in farm yards stables or at other sites close to human activities, so they are more prone to swallow foreign objects that have been carelessly left in their feeding areas. Diagnosis based on the clinical signs may be tentative as all pathognomic signs are not present in all cases, and these pathognomic signs may present in other diseases. Ultrasonography with the scanning of cardiac biomarkers as the most useful laboratory indicators are of great value in the diagnosis of TP as they are positive in all cases even that with cases which do not exhibit all cardiac signs. The treatment is usually not rewarding. Hence, the disease should be better prevented by proper management practices.

## Authors’ Contributions

NEA formulated the research, conducted the study, wrote and revised the manuscript. Final manuscript read and approved by NEA.

## References

[1] Braun U (2009). Traumatic pericarditis in cattle: Clinical, radiographic and ultrasonographic findings. Vet. J.

[2] Grunder H. D, Dirksen G, Grunder H. D, Sto¨ber M (2002). Krankheiten des herzens und des herzbeutels. Innere Medizinund Chirurgie des Rindes.

[3] Reef V. B, McGuirk S. M, Smith B. P (2002). Diseases of the cardiovascular system. Large Animal Internal Medicine.

[4] Radostits O. M, Gay C. C, Hinchcliff K. W, Constable P. D (2007). Diseases of the alimentary tract. In: Veterinary Medicine. A Textbook of the Diseases of Cattle, Horses, Sheep, Pigs and Goats.

[5] Reece W. O (1997). Muscle. Physiology of Domestic Animals.

[6] Boccara G, Pouzeratte Y, Troncin R, Onardet A, Boularan A. M, Colson P, Mann C (2000). The risk of cardiac injury during laparoscopic fundoplication Cardiac troponin I, and ECG study. Acta Anaesth. Scand.

[7] O'Brein P. J, Landt Y, Ladenson J. H (1997). Differential reactivity of cardiac and skeletal muscle from various species in a cardiac troponin I immunoassay. Clin. Chem.

[8] Vasatova M, Pudil R, Horacek J. M, Buchler T (2013). Current applications of cardiac troponin T for the diagnosis of myocardial damage. Adv. Clin. Chem.

[9] Gerber Y, Jaffe A. S, Weston S. A, Jiang R, Roger V. L (2012). Prognostic value of cardiac troponin T after myocardial infarction: A contemporary community experience. Mayo Clin. Proc.

[10] Sharma S, Jackson P. G, Makan J (2004). Cardiac troponins. J. Clin. Pathol.

[11] Rakhit A, Maguire T. C, Wakimoto H, Gehrmann J, Kelly A. R, Michel T, Berul I. C (2001). *In vivo* electrophysiologic studies in endothelial nitric oxide synthase (eNOS)-deficient mice. J. Cardiovasc. Electron.

[12] Flöck M (2004). Diagnostic ultrasonography in cattle with thoracic disease. Vet. J.

[13] Mohamed T (2010). Clinicopathological and ultrasonographic findings in 40 water buffaloes (Bubalusbubalis) with traumatic pericarditis. Vet. Rec.

[14] Jackson P, Cockcroft P (2008). Clinical Examination of Farm Animals.

[15] Kanekeo J. J, Harvey J. W, Bruss M (2008). Clinical Biochemistry of Domestic Animals.

[16] Orro T, Pohjanvirta T, Rikula U, Huovilainen A, Alasuutari S, Sihvonen L, Pelkonen S, Soveri T (2011). Acute phase protein changes in calves during an outbreak of respiratory disease caused by bovine respiratory syncytial virus. Comp. Immunol. Microb.

[17] Hochholzer W, Meissner J, Haaf P, Schaub N, Steuer S, Bassetti S (2011). Incremental value of high-sensitivity cardiac troponin T for risk prediction in patients with suspected acute myocardial infarction. Clin. Chem.

[18] Beda N, Nedospasov A (2005). A spectrophotometric assay for nitrate in an excess of nitrite. Nitric Oxide. Biol. Chem.

[19] Braun U, Goetz M (1994). Ultrasonography of the reticulum in cows. Am. J. Vet. Res.

[20] Braun U, Lejeune B, Schweizer G, Puorger M, Ehrensperger F (2007). Clinical findings in 28 cattle with traumatic pericarditis. Vet. Rec.

[21] Ghanem M (2010). A comparative study on traumatic reticuloperitonitis and traumatic pricarditis in Egyptian cattle. Turk. J. Vet. Anim. Sci.

[22] Sharma S, Gosal N. S, Varun (2012). Idiopathic fibrinous pericarditis in a Nili-Ravi buffalo. Buffalo Bull.

[23] Buczinski S, Francoz D, Fecteau G, DiFruscia R (2010). A study of heart diseases without clinical signs of heart failure in 47 cattle. Can. Vet. J.

[24] Ducharme N. G, Fubini S. L (2004). Surgery of the ruminant forestomach compartments. Farm animal Surgery.

[25] Latimer K. S, Mahaffey E. A, Prasse K. W (2003). Duncan & Prasse's Veterinary Laboratory Medicine: Clinical Pathology.

[26] Jones M. L, Allison R. W (2007). Evaluation of the ruminant complete blood cell count. Vet. Clin. N. Am. Food A.

[27] Saleh M. A, Rateb H. Z, Misk N. A (2008). Comparison of blood serum proteins in water buffaloes with traumatic reticuloperitonitis and sequelae. Res. Vet. Sci.

[28] Gunes V, Atalan G, Citil M, Erdogan H. M (2008). Use of cardiactroponin kits for the qualitative determination of myocardial cell damage due to traumatic reticuloperitonitis in cattle. Vet. Rec.

[29] Meyer D. J, Harvey J. W (2004). Veterinary Laboratory Medicine.

[30] Braun U, Gotz M, Marmier O (1993). Ultrasonographic findings in cows with traumatic reticuloperitonitis. Vet. Rec.

[31] Buczinski S, Rezakhani A, Boerboom D (2009). Heart disease in cattle: Diagnosis therapeutic approaches and prognosis. Vet. J.

[32] Ramakrishna O, Tyagi R P., S, Singh J (1993). Cardiovascular system. Ruminant Surgery.

